# Vof16‐miR‐185‐5p‐GAP43 network improves the outcomes following spinal cord injury via enhancing self‐repair and promoting axonal growth

**DOI:** 10.1111/cns.14535

**Published:** 2024-01-02

**Authors:** Yue Hu, Yi‐Fei Sun, Hao Yuan, Jia Liu, Li Chen, Dong‐Hui Liu, Yang Xu, Xin‐Fu Zhou, Li Ding, Ze‐Tao Zhang, Liu‐Lin Xiong, Lu‐Lu Xue, Ting‐Hua Wang

**Affiliations:** ^1^ Department of Anesthesiology, Institute of Neurological Disease, Translational Neuroscience Center, West China Hospital Sichuan University Chengdu China; ^2^ Department of Anesthesia Operation, The First People's Hospital of Shuangliu District West China Airport Hospital of Sichuan University Chengdu China; ^3^ Laboratory Zoology Department, Institute of Neuroscience Kunming Medical University Kunming China; ^4^ Clinical and Health Sciences University of South Australia Adelaide South Australia Australia; ^5^ Department of Anesthesiology Affiliated Hospital of Zunyi Medical University Zunyi Guizhou China; ^6^ State Key Laboratory of Biotherapy Sichuan University Chengdu Sichuan China

**Keywords:** GAP43, lncRNA volf16, miR‐185‐5p, neurite growth, self‐repair, spinal cord transection

## Abstract

**Introduction:**

Self‐repair of spinal cord injury (SCI) has been found in humans and experimental animals with partial recovery of neurological functions. However, the regulatory mechanisms underlying the spontaneous locomotion recovery after SCI are elusive.

**Aims:**

This study was aimed at evaluating the pathological changes in injured spinal cord and exploring the possible mechanism related to the spontaneous recovery.

**Results:**

Immunofluorescence staining was performed to detect GAP43 expression in lesion site after spinal cord transection (SCT) in rats. Then RNA sequencing and gene ontology (GO) analysis were employed to predict lncRNA that correlates with GAP43. LncRNA smart‐silencing was applied to verify the function of lncRNA vof16 in vitro, and knockout rats were used to evaluate its role in neurobehavioral functions after SCT. MicroRNA sequencing, target scan, and RNA22 prediction were performed to further explore the underlying regulatory mechanisms, and miR‐185‐5p stands out. A miR‐185‐5p site‐regulated relationship with GAP43 and vof16 was determined by luciferase activity analysis. GAP43‐silencing, miR‐185‐5p‐mimic/inhibitor, and miR‐185‐5p knockout rats were also applied to elucidate their effects on spinal cord neurite growth and neurobehavioral function after SCT. We found that a time‐dependent increase of GAP43 corresponded with the limited neurological recovery in rats with SCT. CRNA chip and GO analysis revealed lncRNA vof16 was the most functional in targeting GAP43 in SCT rats. Additionally, silencing vof16 suppressed neurite growth and attenuated the motor dysfunction in SCT rats. Luciferase reporter assay showed that miR‐185‐5p competitively bound the same regulatory region of vof16 and GAP43.

**Conclusions:**

Our data indicated miR‐185‐5p could be a detrimental factor in SCT, and vof16 may function as a ceRNA by competitively binding miR‐185‐5p to modulate GAP43 in the process of self‐recovery after SCT. Our study revealed a novel vof16‐miR‐185‐5p‐GAP43 regulatory network in neurological self‐repair after SCT and may underlie the potential treatment target for SCI.

## Graphical abstract

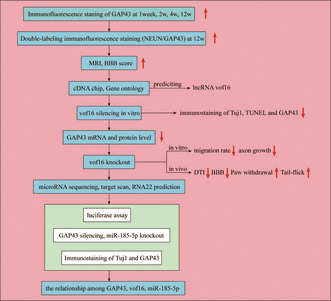

## 1 INTRODUCTION

Spinal cord injury (SCI) occurs commonly in younger population of the modern society,^1^ usually leading to permanent disability characterized by paralysis and loss of sensation to peripheral stimuli, multiple metabolic, and systemic alterations associated with the autonomic nervous system dysfunction.^2^ Spinal cord transection (SCT) is the most severe type of spinal cord injury, which put patients at a high risk for permanent disability. After SCT, the injured descending and ascending axons fail to regenerate in adult mammals, resulting in severe acute functional deficiencies.^3^ During the past decades, nervous and non‐nervous tissue transplantation, gene therapy,^4^ cell transplantation,^5^ and cell transplantation in combination with neurotrophic factors (NTFs)^6^ were the focuses in pre‐clinical research of SCI. However, there are still limited clinical therapies available for effective management. Nevertheless, SCI self‐repair has been found in humans and experimental animals^7^ and locomotor function of rats is partially recovered in 7 days after SCI.^8^ Some findings have shown that after severe SCI, prominent functional recovery appear without the regeneration or maintenance of direct projections from the brain past the lesion site, which is mediated by the reorganization of descending and propriospinal connections.^9^, ^10^, ^11^, ^12^ And such recovery is attributed to axon plasticity in the form of sprouting of spared and/or injured axon populations. Previous study also pointed out that neurites loss of neurons may be one of the factors that cause motor dysfunction of hind limbs.^13^ However, the regulatory mechanisms underlying the spontaneous locomotion recovery after SCI injury are uncertain and need to be further investigated.

Self‐reparative potential after injury is often associated with factors regulated at the gene and protein levels involving in facilitating neurite outgrowth.^14^, ^15^ Studies have shown that during normal development of nervous system or regeneration of injured nerves, growth‐associated protein 43 (GAP43) synthesized in growth cones is necessary for the guidance and elongation of growing axons.^16^, ^17^ Moreover, it also has neuroprotective effects and can regulate the proliferation of neuronal precursors.^18^, ^19^ This protein is strictly conserved among vertebrates^20^ and plays a role in contributing to regulating and modulating neurite outgrowth, axon regrowth, neuronal plasticity, and synaptogenesis following SCI.^14^, ^21^ However, there is no study particularly focusing on GAP43 and its interacted genes involved in SCI self‐repair.

Extensive work has been done to study the spatial and temporal gene expression changes by screening lncRNAs, of which a small proportion may play a vital role in the pathophysiology of SCI.^3^, ^22^ It was reported that lncRNAs can regulate gene expression in *cis* or *trans* way via recruitment of proteins or molecular complexes to specific loci, serve as scaffolds to form cytoplasmic or nuclear complexes, and pair with other RNAs to trigger post‐transcriptional regulation.^23^, ^24^ Intriguingly, lncRNAs could function as competing endogenous RNAs (ceRNAs) which share miRNA recognition elements (MREs) to compete for miRNA binding and regulate each other.^25^, ^26^ Furthermore, it has been documented that a crosstalk exists between lncRNAs and miRNAs, which participates in varieties of disease processes, such as melanoma, prostate cancer, glioblastoma multiforme (GBM), solid tumors, and hematopoietic malignancies.^27^, ^28^, ^29^ However, little is known whether this crosstalk underlay the self‐repair potential after SCT. Thus, we put forward a hypothesis that certain lncRNAs might be involved in the promotive activity for the spontaneous neurological function recovery after spinal cord injury. The associated in‐depth mechanisms were investigated in this study.

## 2 MATERIALS AND METHODS

### 2.1 Animal care

All the adult female Sprague–Dawley (SD) rats weighing 200–250 g were purchased from Animal Center of Sichuan University (RRID: RGD_734476). Guidelines from NIH for laboratory animal care and safety were strictly followed. Animals were housed in cages with a 12‐hour (h) light/dark cycle, accessible to food and water ad libitum throughout the study. Aseptic environment was maintained during whole surgical procedures. Vof16 and miR‐185‐5p knockout rats were established in Cyagen Biosciences (Cyagen, Guangzhou, China). All experimental procedures involving animals were conducted following institutional animal care guidelines and approved by Administration Committee of Experimental Animals, Sichuan Province, China.

### 2.2 Spinal cord transection (SCT) model establishment

In our study, female rats were utilized as urinary dysfunction is usually induced after spinal cord injury. The incidence of urethral infection in female rats is relatively low, and it is easier to squeeze urine out of the bladder. Sixty adult female SD rats (200–250 g) were randomly divided into normal (*n* = 10), sham (*n* = 20), and SCT (*n* = 30) groups. The procedure was processed as previous described.^30^ Briefly, rats were anesthetized with 3% isoflurane and immobilized in the prone position, and then they underwent a skin incision and paravertebral muscle dissection. The laminectomy was performed at thoracic vertebra level between T9 and T11 to expose T10 spinal segment. Afterward, the spinal cord was transected completely by micro‐scissors at the T10 level. To ensure the completeness of transection, the transected site should be assured by lifting the cut ends with small forceps. Sham‐injured animals underwent laminectomy only with no further spinal cord damage. Finally, the surgery incision was disinfected and sutured. After SCT, animals were placed in warm condition to maintain their body temperature. All rats received an intramuscular injection of penicillin (160,000 U/mL/day, Harbin Pharmaceutical Group) daily for consecutive 7 days after the surgery. Their bladders were manually massaged three times per day to help urinating.

### 2.3 Tissue preparation

According to the different phases of experiments, the preparation of tissue harvest points is different at 1, 2, 4, and 12 weeks after SCT. Briefly, 1 cm spinal cord (0.5 cm above and below the traverse section) was immediately harvested from animals of each group and stored at −80°C for further use. For immunofluorescence staining, the tissues were harvested after intracardiac perfusion with 0.9% physiological saline followed by 4% paraformaldehyde (Cat# 142287, Beyotime, Shanghai, China) at 4°C, pH 7.4.

### 2.4 Magnetic resonance imaging (MRI)

A 4.7‐T/40 cm magnetic resonance scanner (Bruker Biospec 70/30, Ettlingen, Germany) was used to detect spinal cord injury as previously described.^31^ All rats were anesthetized with 2% isoflurane (Cat# R580, RWD, Shenzhen, China) in air/O_2_ (4/1). The body temperature was maintained at 37°C using a heating blanket and monitored with a rectal temperature probe. The imaging protocol for all rats included a T2 spin‐echo sequence (rare factor: 8; TR/TE: 5000 msec/50 msec, in‐plane resolution of 117 μm × 98 μm; field of view: 3.0 cm × 2.5 cm). Eight sagittal slices at thickness of 0.75 mm were acquired from each rat.

### 2.5 Immunofluorescence staining

Immunofluorescence staining of growth‐associated protein 43 (GAP43) or NEUN was performed to detect the change of expression at 1, 2, 4, and 12 weeks after SCT. In brief, after the harvested spinal cord tissues (1 cm long, 0.5 cm above and below the lesion site) were dehydrated overnight by 30% sucrose, they were cut at 10 μm thickness by freezing microtome (Leica CM1900, Germany). The sections were rinsed three times by 0.01 M PBS (Cat# BL551A, Biosharp, Hefei, China), and then incubated with 5% goat serum (Cat# SL038, Solarbio, Beijing, China) for 30 minutes (min) at 37°C to block non‐specific binding. Next, the sample sections were incubated with anti‐GAP43 antibody (1:50, Mouse, Santa Cruz Biotechnology Cat# sc‐135915) or anti‐NEUN (1:200, Rabbit, GeneTex Cat# GTX37604) for over 18 h at 4°C. After rinsing the sections with PBS three times for 5 min, secondary antibodies of Cy3 (1:100, goat anti‐mouse, ZSGB‐Bio, Cat# ZF‐0312) or dylight 488 (1:200, goat anti‐rabbit, Abbkine, Cat# A23020) were used and incubated at room temperature for over 18 h. Negative control underwent the same procedures without using primary antibody. Moreover, cell nuclei were determined by DAPI‐fluoromount. The sections were observed by BX‐51 microscope (Olympus, Tokyo, Japan). In the graft and the host spinal cord mass located at the mouth side of the lesion, the quantification was performed at the following distances: 0–250, 250–500, 500–750, 750–1000, and 1000–1250 μm. Moreover, the fluorescence intensity was calculated using Image‐Pro Plus 6.0 software (MediaCybernetics, Silver Spring, MD, USA).

### 2.6 Basso, Beatlie, and Bresnahan (BBB) score

Hind limb function of the rats was assessed on 1, 2, 4, 6, 8, 10, and 12 weeks after surgery with a Basso, Beattie and Bresnahan open field locomotion rating scoring system (BBB score).^32^ All measurements were performed under a double‐blinded procedure. Each animal was evaluated for 4 min and assigned an operative‐defined score for each hindlimb. The average score was determined by three independent researchers who were blind to the different experimental treatments.

### 2.7 Microarray analysis

The rat long non‐coding RNA (lncRNA) array was designed to profile lncRNAs and protein coding genes.^33^ The total RNA extract from injury spinal cords (about 0.5 cm distance from the transaction point) using Trizol (Invitrogen, Carlsbad, CA). Sample labeling and array hybridization were performed according to the previous description by using Gene Chip WT Terminal Labeling and Controls Kit as well as the Gene Chip Hybridization, Wash, and Stain Kit.^34^ The synthesized double‐stranded complementary DNA (cDNA) were labeled and hybridized to the Affymetrix Gene Chip Rat Gene 2.0 ST Array Microarray (Arraystar, Rockville, MD). After hybridization and washing, processed slides were scanned with Axon Gene Pix 4000B microarray scanner (Molecular Devices, Sunnyvale, CA). A *p*‐value was calculated using the paired *t*‐test. The threshold set for up‐regulated and down‐regulated genes was a fold change >¼ 2.0 and a *p*‐value < ¼ 0.05.

### 2.8 LncRNA profiles analysis

LncRNA and mRNA expression profiles were compared, the significance and false discovery rate (FDR) were calculated by using the adjusted *F*‐test with the random variation model.^35^ The LncRNA profiles were screened out by the UCSC and NCBI analysis. The annolnc website (http://annolnc.cbi.pku.edu.cn/) was used to predict the possible target protein of lncRNA, while the DAVID website was used to analysis those proteins as GO and pathway.

### 2.9 Clustering and sequencing

The clustering of the index‐coded samples was performed on acBot Cluster Generation System using TruSeq PE Cluster Kitv3‐cBot‐HS (Illumia) according to the manufacturer's instructions.

### 2.10 Gene ontology (GO) and pathway analyses

Gene ontology analysis provides a controlled vocabulary to describe genes and gene products which attribute in any organism (http://www.geneontology.org). This ontology covers three domains: molecular functions, cellular components, and biological processes. Fisher's exact test was applied to find if there is more overlap between DE list and GO annotation list than would be expected by chance. The *p*‐value denotes the significance of GO term enrichment in the DE genes (*p*‐value ≤ 0.05 is recommended).

Pathway analysis is used to map genes to KEGG pathways. The *p*‐value (Fisher *p*‐value, EASE score, or hypergeometric *p*‐value) denotes the significance of the pathway correlations (*p*‐value ≤ 0.05 is recommended).

### 2.11 The construction of vof16 and miR‐185‐5p knockout rats

Vof16 knockout and miR‐185‐5p knockout rats were constructed in Cyagen Biosciences Inc. CRISPR/Cas‐mediated genome engineering technology was applied to construct vof16 or miR‐185‐5p knockout rats. Briefly, sgRNA and Cas9 mRNA were synthetized firstly, and then were injected into 150 fertilized eggs which were subsequently transplanted into 5 pseudo pregnant rats. Genomic identification for vof16 showed that the molecular weight of the wild type was 671 bp, and the homozygote was 570 bp, while the genomic identification for miR‐185‐5p confirmed that the molecular weight of the wild type was 750 bp, and the homozygote was 520 bp. Finally, vof16 or miR‐185‐5p knockout rats were constructed successfully.

### 2.12 Genotype identification

Vof16 knockout and miR‐185‐5p knockout rats were constructed in Cyagen Biosciences Inc. The tail tips were collected and then the rats' genomic DNA was extracted using Transgen's genomic DNA extraction kit (ee101‐12), and the DNA sequencing primers for vof16 and miR‐185‐5p were as following:

Rat vof16‐F: 5′‐ AGTTTGTCCGAGTGATGGGAATACAC ‐3′
Rat vof16‐R: 5′‐ GACTCCGTGAGCCATTTATCATTCTG ‐3′
Rat miR‐185‐5p‐F: 5′‐ CTGATGTGCTCAGGGTGTTGACC ‐3′
Rat miR‐185‐5p‐R: 5′‐ GCTGCTGATGTTAGGGAGGAGGC ‐3′.

Additionally, the reaction was performed in mixtures system included 10 μL PCR master mix 0.6 μL upstream primers, 0.6 μL downstream, 3 μL DNA template, and 5.8 μL water. The thermal cycling conditions were performed as: initial denaturation at 94°C for 5 min, and 35 cycles of denaturation at 94°C for 30 s, annealing at 60°C for 30 s, with elongation at 72°C for 30 s, followed by elongation at 72°C for 5 min and the storage at 12°C. Afterward, genotype was detected by agarose gel electrophoresis system. After genotype identification, the knockout rats were subjected to SCT operation, and the corresponding experiments were performed on vof16 or miR‐185‐5p knockout rats and their wild‐type mates.

### 2.13 Primary spinal cord and cortical neurons culture

The vof16 +/+, +/−, and −/− rats were used for culturing primary spinal cord and cortical neurons, while the miR‐185‐5p knockout rats was only applied for the culture of spinal cord neurons. Briefly, the cortexes and spinal cord of rats were harvested and minced at the size of 1 mm^3^ and isolated by 0.25% trypsin for 10 min at 37°C; then, the tissues were eluted with the 10% BSA (Cat# A8020, Solarbio, Beijing, China). Subsequently, the cells were collected by centrifugation at 1000 rpm for 10 min, resuspended by 10% BSA, and plated into 6‐well plates (Corning, USA) at a density of 2–5 × 10^5^ cells/ml. After incubation for 4 h at 37°C with 5% CO_2_, the culture medium was replaced by Neurobasal added with 2% B27. The culture medium was changed the next day; then one‐half change was performed every 3 days. The adherent cells were round, transparent and sprout tiny neurite at the 3 days of culture. The cell body of neurons were plumped, the cytoplasm was transparent, and refraction was good, the nucleus was round, dendritic axon was matured at 7 days of culture, then the cells could be taken to immunocytochemical staining for identifying the purity of neurons.

### 2.14 RNA interference

For inhibition of endogenous vof16 expression, spinal cord neurons were transfected with Ribo™ lncRNA Smart Silencer (100 mM, #cat: lnc3N0000001‐1‐5, Ribobio, Guangzhou, China; http://ribobio.bioon.com.cn/) using riboFECT CP Reagent (Ribobio) following the manufacturer's instructions at the time of plating. After approximately 7 days of culturing, lncRNA Smart Silencer was transfected into spinal cord neurons following the manufacturer's instructions provided by Ribo company. Briefly, at about 60%–70% confluence, neurons were incubated with a serum‐free medium as above for 2 h at 4°C, then incubated with 5% CO_2_ at 37°C. Spinal cord neurons with transfected successfully indicated by CY3 (red fluorescent) and were photographed on the 3rd and 5th days after transfection.

### 2.15 Transfection of miR‐185‐5p mimics/Inhibitor into cortical neurons

To detect the role of miR‐185‐5p on the neurite outgrowth and cell apoptosis in spinal cord neurons, miR‐185 mimic/inhibitor (provided by RiboBio, Guangzhou, China) were transfected into cortical neurons after culturing for 3 days. The transfection system of miR‐185‐5p was the mixture of 1x ribo FECTTMCP Buffber, 100 ng/μL ribo FECTTMCP Regent and miR‐185‐5p mimic (80 nM) or miR‐185‐5p inhibitor (100 nM), the mixture was added drop‐wise to the appropriate wells, respectively. The Inversed Fluorescent Microscope (Leica, Wetzlar, Germany) was used to evaluate the transfection efficiency.

### 2.16 Immunofluorescence

For immunofluorescence studies in vitro, the cells were grown in 24‐well plates as described previously. The samples used in Immunofluorescence straining were collected on the 3rd days after transfection The anti‐GAP43 antibody (1:50, Mouse, Santa Cruz Biotechnology, Cat# sc‐135915) or Neuronal Class III β‐Tubulin (Tuj1) (1:400, Mouse, Millipore, Cat# MAB1637) or Tuj1 (1:400, rabbit, Millipore, Cat# T2200) and the secondary antibodies of dylight 488 (1:100, goat anti‐rabbit, Abbkine, Cat# A23220) or Cy3 (1:200, goat anti‐mouse, Jackson ImmunoResearch Labs Cat# 015‐160‐006) were provided. Five fields were taken to measure cell size and number as well as neurite length of spinal cord neurons using Leica DMI6000B (LAS AF system). A fluorescence microscope was used to observe the tissue sections. ImageJ was used to examine the fluorescence intensity.

### 2.17 TUNEL assay

Terminal‐deoxynucleotidyl Transferase Mediated Nick End Labeling (TUNEL) staining in cultured spinal cord neurons were performed. Briefly, the samples were collected on the 3rd days after transfection, cultured neurons were fixed with 4% paraformaldehyde (Cat# 142287, Beyotime, Shanghai, China) for 20 min at room temperature, then rinsed with PBS (Cat# BL551A, Biosharp, Hefei, China) for 20 min. Subsequently, cells incubated with TUNEL reaction mixture (Cat# Rs‐11684817910, In situ Cell Death Detection Kit, Roche Molecular Biochemicals, Mannheim, Germany) for 1 h at 37°C, then rinsed with PBS for 3 times and finally incubated with DAPI (Cat# C1005, Beyotime Biotechnology, Shanghai, China) for 5 min. Five fields were acquired, and apoptosis was quantified by determining the percentage of TUNEL/DAPI with Leica AF6000 DMI6000B (LAS AF system).

### 2.18 Quantitative real‐time polymerase chain reaction (qRT‐PCR)

Quantitative reverse transcription PCR was performed as previously described.^36^ Total RNA of caudal and rostral spinal cords, tissue harvest at 12 weeks after SCT, cultured spinal cord neurons (tissue harvest on the 3rd days after transfection) was extracted using the Trizol reagent (Invitrogen, Life Technologies, Carlsbad, CA, USA) and reversely transcribed to cDNA with the Revert Aid (Invitrogen). Then,  qRT‐PCR analysis was performed to analyze the level of vof16, GAP43, miR‐330‐3p, miR‐351‐5p, miR‐28‐5p, miR‐30c‐1‐3p, miR‐185‐5p, miR‐451‐5p, miR‐434‐3p, and miR‐6318. Information on primer sequences was follows: vof16 sense: 5′ TGCTTGGCCTCAGAACATCT 3′; antisense: 5′ GTCAGGAAAACCTAGTCACAT 3′; GAP43 sense: 5′ TGAGAAGAACCAAACAGGTTG 3′; antisense: 5′ CTTTGAGCTTTTTCCTTGTT 3′; miR330‐3p (GeneCopoeia, Lnc. HmiRQP0970); miR351‐5p (GeneCopoeia, Lnc. HmiRQP1250); miR28‐5p (GeneCopoeia, Lnc. HmiRQP0362); miR30c‐1‐3p (GeneCopoeia, Lnc. HmiRQP0394); miR185‐5p (GeneCopoeia, Lnc. HmiRQP0247); miR451‐5p (GeneCopoeia, Lnc. HmiRQP0509); miR434‐3p (GeneCopoeia, Lnc. HmiRQP1002); miR6318‐5p (GeneCopoeia, Lnc. HmiRQP2815); U6 (GeneCopoeia, Lnc. HmiRQP9003); GAPDH sense: 5′ GACATGCCGCCTGGAGAAAC 3′; antisense: 5′ AGCCCAGGATGCCCTTTAGT 3′. PCR amplification was performed as follows: (1) initial denaturation (1 cycle, 95°C for 3 min); (2) denaturation (40 cycles 95°C for 15 s); (3) annealing (40 cycles 60°C for 10 s); (4) amplification (40 cycles, 51°C for 30 s, 60°C for 40 s). Data were analyzed using a comparative critical threshold (Ct) method where the relative expression of target normalized to the amount of endogenous control.

### 2.19 Western blot

Western blot assay was descried previously.^30^ Briefly, after spinal cord neurons which were transfected by lncRNA volf16 smart silence 3 days, the cells were lysed in RIPA buffer (Beyotime) including cocktail pill (50:1 V/V). A volume with 100 μg of total protein was resolved in 15% SDS–polyacrylamide gel from cell suspension. Then electrophoresis was performed at 60 V for 30 min, then 100 V for 1.5 h to separate the proteins, and proteins were transferred onto PVDF membranes at 350 mA for 4 h, followed by blocking with TBST, containing 5% fat‐free milk for 1 h at room temperature. After that the membranes were incubated with the primary antibodies, GAP43 antibody (1:1000, mouse, Santa Cruz Biotechnology, Cat# sc‐33705) and β‐actin (1:5000, rabbit, Santa Cruz Biotechnology, Cat# sc‐47778 HRP) in TBS overnight at 4°C. After the membranes were repeatedly rinsed in TBST four times, they were incubated for 1.5 h in the secondary antibody (goat anti‐rabbit, goat anti‐mouse, 1:5000, Abclonal). Finally, the membranes were rinsed 4 times in TBST and developed in Alpha Innotech (BIO‐RAD) with ECL. The Western blot bands of GAP43 in comparison to ß‐actin as internal control were analyzed by calculating the ratio of their optical density to determine the difference. The relative density of the protein bands was analyzed by ImageJ software (FIJI, RRID: SCR_002285) and normalized to that of ß‐actin.

### 2.20 Wound healing test

Wound healing tests were used to measure cell migration in spinal cord and cortical neurons. The horizontal line was evenly crossed by a marker pen with a ruler, and the well was 0.5–1 cm at approximately once each time. In brief, neurons were plated into the wells at a density of 1 × 10^6^ cells/ml. The next day, straight scratches which crossed the 60‐mm culture dish were created with a p200 pipette tip, simulating a wound. Then cells were washed three times with the culture medium and added to the fresh medium and incubated at 37°C and 5% CO2. Images were acquired at 0, 24, 30, 48, and 72 h, respectively under Nikon Eclipse Ti‐S fluorescence microscope with a random view of five scratch areas. The distance of cell migration equals the difference between the shortest distance of 0 h and that of 24, 30, 48, and 72 h.

### 2.21 Diffusion tensor imaging (DTI) examination

Diffusion tensor imaging was used to noninvasively and longitudinally track spinal cord neural regeneration progress in SD rats at 12 weeks after spinal cord transection. DTI was performed using SE echo‐planar imaging (EPI) (9500 ms TR, 70 ms TE, 256 × 256 matrix size, 250 mm FOV, 2 mm section thickness with no gaps). Images were acquired with no diffusion encoding (*b* = 0 s/mm^2^) and with 32‐direction diffusion encoding (*b* = 1000 s/mm^2^ for each direction) and were corrected for cardiac and head motion artifacts. DTI data were then sent to a workstation (Philips extended MR workspace 2.6.3.2, Philips Medical Systems) and processed using DTI fiber tracking software for DTI quantitative analysis. Fiber tracts were reconstructed by the fiber assignment continuous tracking (FACT) method. Volumes of fiber tracts were counted. All ROIs on tensor color maps and FA and DTI fiber counts were independently drawn or measured by two neuroradiologists. To reduce the random variability in the measurements, each value was averaged from three different measurements.

### 2.22 Paw withdrawal thresholds (PWT)

Spinal cord injury‐induced mechanical hyperalgesia was evaluated by measuring hind paw withdrawal thresholds to an increasing pressure stimulus by using an analgesy‐meter (37215; Ugo Basile, Comerio, Italy) with a wedge‐shaped probe. The hind paw of rats was placed on a small plinth under a cone‐shaped pusher with a rounded tip so that the force could be applied to the paw. Then, the operator depressed a pedal switch which exerted the force to start the mechanism. When the rat struggled, the operator released the pedal and read off the scale of the force.

### 2.23 Tail‐flick test (TFL)

For tail‐flick latency test, rats were individually placed on an elevated iron mesh in a clear plastic cage and were adapted to the test environment for at least 30 min. The infrared source was applied to the tail of each rat. Only immediate and robust tail responses from the stimulus were recorded. The time of 22 s was selected for testing as the upper cutoff time.

### 2.24 Bioinformatics prediction and detection

Gene co‐expression network was established in KangChen Bio‐tech (Shanghai, China) according to the normalized signal intensity of specific expression LncRNAs and miRNAs.^37^ MiRanda and TargetScan (http://www.targetscan.org/vert_72/) were applied to predict the target miRNAs of vof16.^38^ TargetScan and miRDB (http://www.mirdb.org/) were used to predict the target miRNAs of GAP43 and predict the target GAP43 of miRNAs. Additionally, RNA22 (https://cm.jefferson.edu/rna22/Interactive/.venny2.1) was used to predict the binding site between lncRNA and miRNA, whereas Venny 2.1.0 (http://bioinfogp.cnb.csic.es/tools/venny/) was used to draw Venn diagrams. Both were used to screen for the most effective target miRNAs which were further verified by qRT‐PCR.

### 2.25 Luciferase assay

Luciferase can be used as a reporter gene to measure the activity of promoters, or/and the transfection efficiency. Gap43 3′UTR luciferase plasmids and the corresponding Mut plasmids were generated by RiboBio (GuangZhou, China). Vof16‐mut was constructed by GeneCopeia Company (GuangZhou, China). The pmiR‐RB‐REPORT™ Dual‐luciferase‐expressing vector contained hRlucc DNA encoding *Renilla* luciferase as a reporter and hLucc DNA encoding firefly luciferase as an internal control. Constructs of WT luciferase plasmid contained the full‐length 3′‐UTR of GAP43 mRNA, and the mutant plasmids contained a 3′UTR mutation (mutated from “TCTCTCC” to “AGAGAGG”) and vof16 mut (mutated from “CCTTTCTCTC” to “GGAAAGAGAG”) could effectively abrogate the binding of GAP43 and vof16 to miR‐185‐5p. The constructs were confirmed by NotI and XhoI restriction enzyme digestion and sequencing. Then, 293Tα cells (4 × 10^3^ cells/well) were seeded into triplicate wells of 96‐well plates 1 day before transfection. Afterward, the mixture of 3′UTR luciferase plasmids of GAP43 or vof16 plasmid or control reporter plasmid (100 ng/mL, Guangzhou RiboBio, China) and miR185‐5p/mimic‐nc (final concentration 80 nM) were transfected into 293Tα cells by using SuperFectinTM II in Vitro DNA Transfection Reagent (Pufei Biotech, China). Forty‐eight hours after transfection, luciferase activity was measured with Dual‐Luciferase Reporter Assay Kits (Promega, E1910). The fluorescence value of Renilla fluorescence/firefly is the final relative luciferase activity.

### 2.26 Statistical analysis

Statistical analysis was performed by using SPSS 21.0 software (IBM SPSS Statistics). The Shapiro–Wilk test was used to assess data distribution. For data conforming to the normal distribution, one‐way ANOVA, Student–Newman–Keuls (SNK) test, or independent sample *t*‐test was used for statistical comparison. For the data that did not conform to the normal distribution, we analyzed it through non‐parametric equivalence, such as Mann–Whitney *U*‐test or Bonferroni post‐mortem test. Statistical comparisons were carried out among more than two groups using one‐way ANOVA with Student–Newman–Keuls (SNK) post hoc test, while the independent‐sample *t*‐test was used to compare the data between two groups. The Mann–Whitney *U*‐test was used instead when the variance was not equal, then the Bonferroni post hoc test was performed if the variances were equal. The experimental data were expressed as mean ± standard deviation (SD). The correlation analysis was applied to analyze the relationship between BBB score and GAP43 intensity. Results were considered statistically significant at *p* < 0.05. Figures were prepared using Adobe Photoshop CS6 (Adobe Systems) and Prism 6.0.

## 3 RESULTS

### 3.1 Limited neurological recovery in SCT rats is associated with GAP43 time‐dependent increase and a group of undetermined lncRNA

**FIGURE 1:**
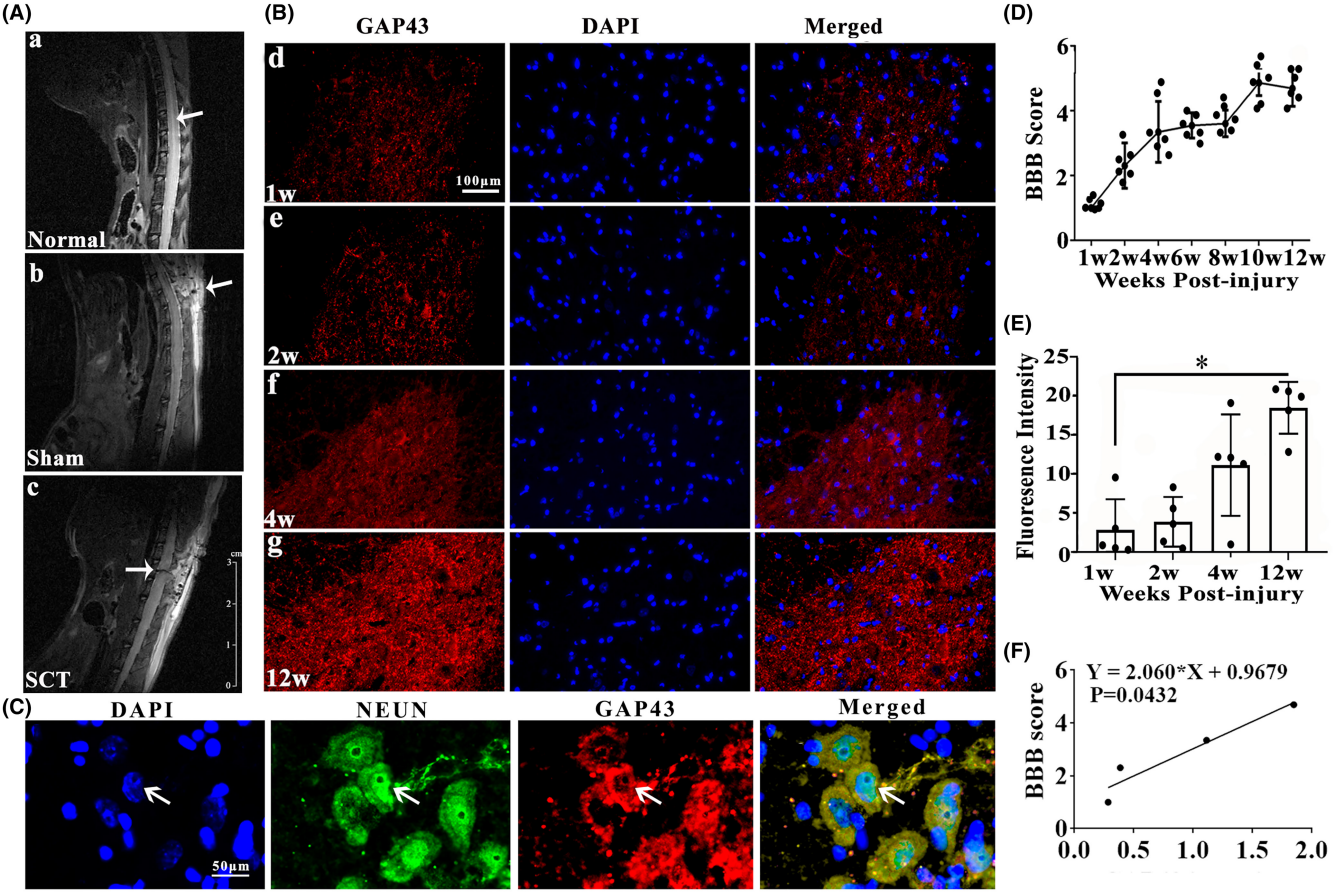
Expression of GAP43 after SCT. (A‐a–c) MRI images of spinal cord from the normal, sham, and SCT rats. The white arrow expresses the injury site, *n* = 3/group. (B‐d–g) Immunofluorescence staining of GAP43 at 1, 2, 4, and 12 weeks after SCT in spinal cord. GAP43 was stained by red fluorescence; the nuclei were stained by DAPI (blue color), *n* = 10/group. Scale bar = 100 μm. (C) Double immunostaining of GAP43 (red) and NEUN (green) in spinal cord at 12 weeks after SCT, *n* = 10/group. Scale bar = 50 μm. White arrow points at the positive cells. (D) BBB score at 1, 2, 4, 6, 8, 10, and 12 weeks in SCT rats post‐injury, *n* = 7/group. (E) Quantitative histograms of fluorescence intensity of GAP43 at 1, 2, 4, and 12 weeks after SCT. (F) The correlation analysis between BBB score and GAP43 intensity, *r* ^2^ = 0.9155, *p* < 0.05. All data were presented as mean ± SD. Data were analyzed using one‐way ANOVA followed by Student–Newman–Keuls (SNK) post hoc test for multiple comparisons, *p < 0.05 and **p < 0.01. BBB score, Basso, Beatlie, and Bresnahan score; SCT, spinal cord transection.

The MRI which was applied to confirm the successful operation of SCT, obviously showed the complete transection of T10 spinal segment (Figure 1A‐a–c). As reflected by BBB scores, SCT rats exhibited motor dysfunction in hind limbs at 1 week after injury, but the motor functions gradually improved over time indicated by gradually increased scores, especially at 12 weeks after the SCT (Figure 1D). Growth‐associated protein 43 (GAP43), also known as neuromodulin, is an axonal membrane protein involved in the extracellular growth of nerve cell, synapse development and formation, and nerve cell regeneration.^39^ The results of immunofluorescence staining demonstrated that fluorescence intensity of GAP43 at 12 weeks is significantly stronger than 1 week after injury (Figure 1B‐d–g,E, *p* < 0.05). The significant induction of GAP43 positive signals at 12 weeks after SCT was also confirmed in the representative images of double staining detection of GAP43 (red) and NEUN (green) (Figure 1C). Interestingly, we found that BBB score and GAP43 intensity at 1, 2, 4, and 12 weeks have a strong positive correlation after SCT (Figure 1F, *r* ^2^ = 0.9155, *p* < 0.05).

**FIGURE 2:**
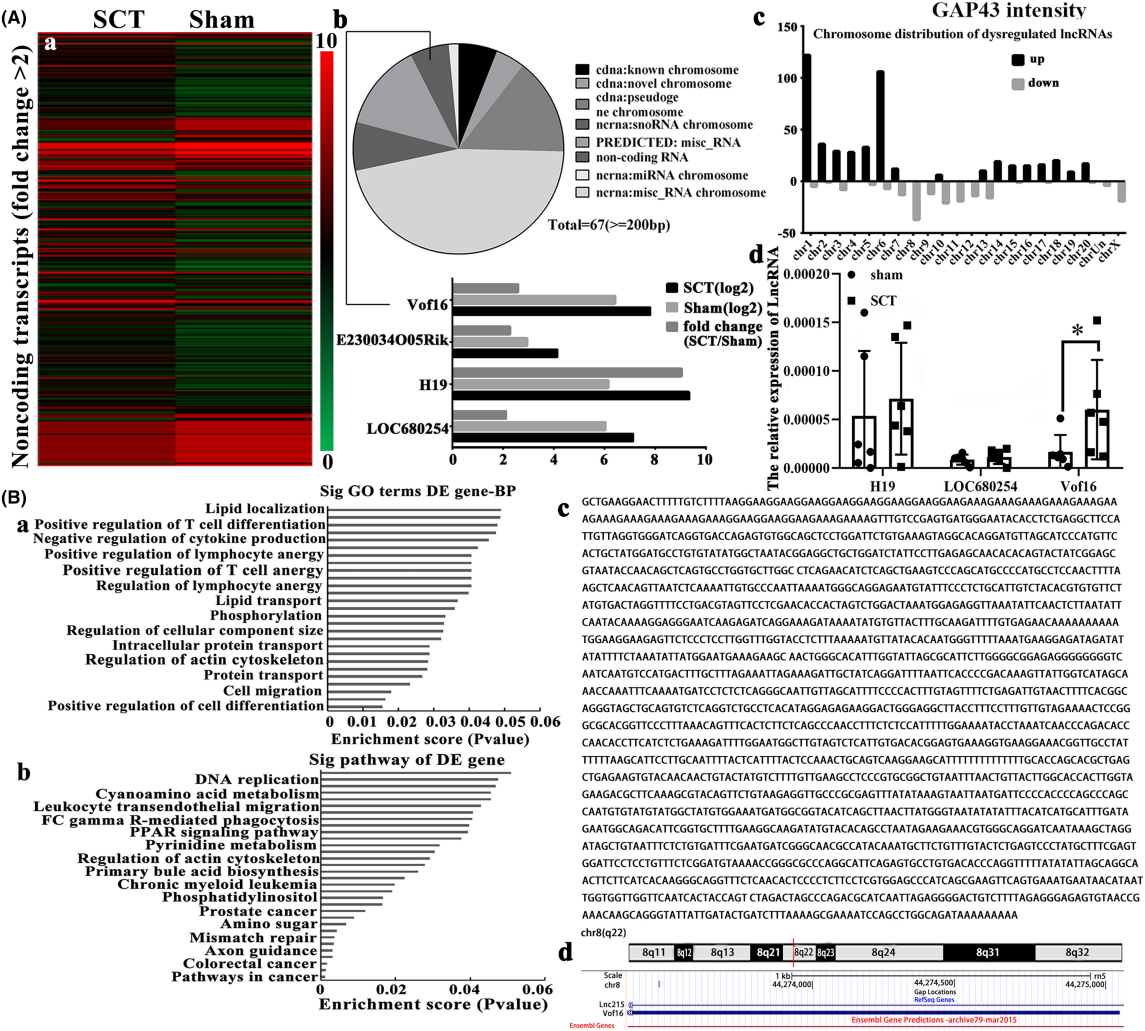
Bioinformatics analysis of lncRNA vof16 after SCT. (A‐a) Heat map and hierarchical clustering analysis of 675 lncRNAs that were differentially expressed (fold change >2) in rat spinal cord between the SCT and sham. A color tag represents the expression levels of genes, red spectrum denotes up‐regulated genes, green for down‐regulated ones. (A‐b) Pie chart of GO analysis for selecting the target lncRNA to GAP43 and the fold change of four lncRNAs including vof16, E230034O05Rik, H19, and LOC680254 by PCR verification, *n* = 6/group. (A‐c) Chromosome distribution of up‐ and down‐regulated lncRNAs in different chromosomes, *n* = 6/group. (A‐d) The bar charts of the relative expression of lncRNA among H19, LOC680254 and vof16 in the groups of sham and SCT, *n* = 6/group. (B‐a, b) The significant terms of Vof16 lncRNA in GO and KEGG pathway analysis using DAVID website (*p* < 0.05), *n* = 6/group. (B‐c, d) The full sequence and scheme of vof16 in rat genome. The data of PCR results were analyzed by independent sample *t*‐test and presented as mean ± SD, *p < 0.05. GO, gene ontology; KEGG, Kyoto encyclopedia of genes and genomes; SCT, spinal cord transection.

In order to further explore the regulatory mechanism of GAP43, hierarchical clustering was performed to screen the differentially expressed lncRNAs between the sham and SCT samples (Figure 2A‐a). Those lncRNAs with highly similarity and shorter than 200 nucleotides were excluded, then we found only four lncRNAs were prominently altered which were vof16, E230034O05Rik, H19, and LOC680254 (Figure 2A‐b). The chromosome distribution showed that the number of up‐regulated and down‐regulated lncRNAs were in each chromosome (Figure 2A‐c). To validate the outcome of microarray analysis, qRT‐PCR was carried out and it demonstrated that the relative expression of vof16 lncRNA was notably up‐regulated in the injured spinal cord compared with the sham group (Figure 2A‐d, *p* < 0.05). Furthermore, KEGG pathway and Gene Ontology (GO) analysis were employed to predict potential lncRNAs using DAVID (https://david.ncifcrf.gov/), and vof16 lncRNA exhibited association with axon guidance pathway (Figure 2B‐a,b). Subsequently, we performed the RACE to determine the full sequence of vof16 (Figure 2B‐c) through the UCSC Genome Browser (http://genome.ucsc.edu/). We then found that vof16 is a ~2107‐nucleotide transcript comprising one exon and located on chromosome 8q22. Besides, when compared with rat genome, the sequence of vof16 overlaps with lnc215 (Figure 2B‐d).

### 3.2 Interfering vof16 lncRNA could reduce axon length and increase apoptosis in spinal cord neurons

**FIGURE 3:**
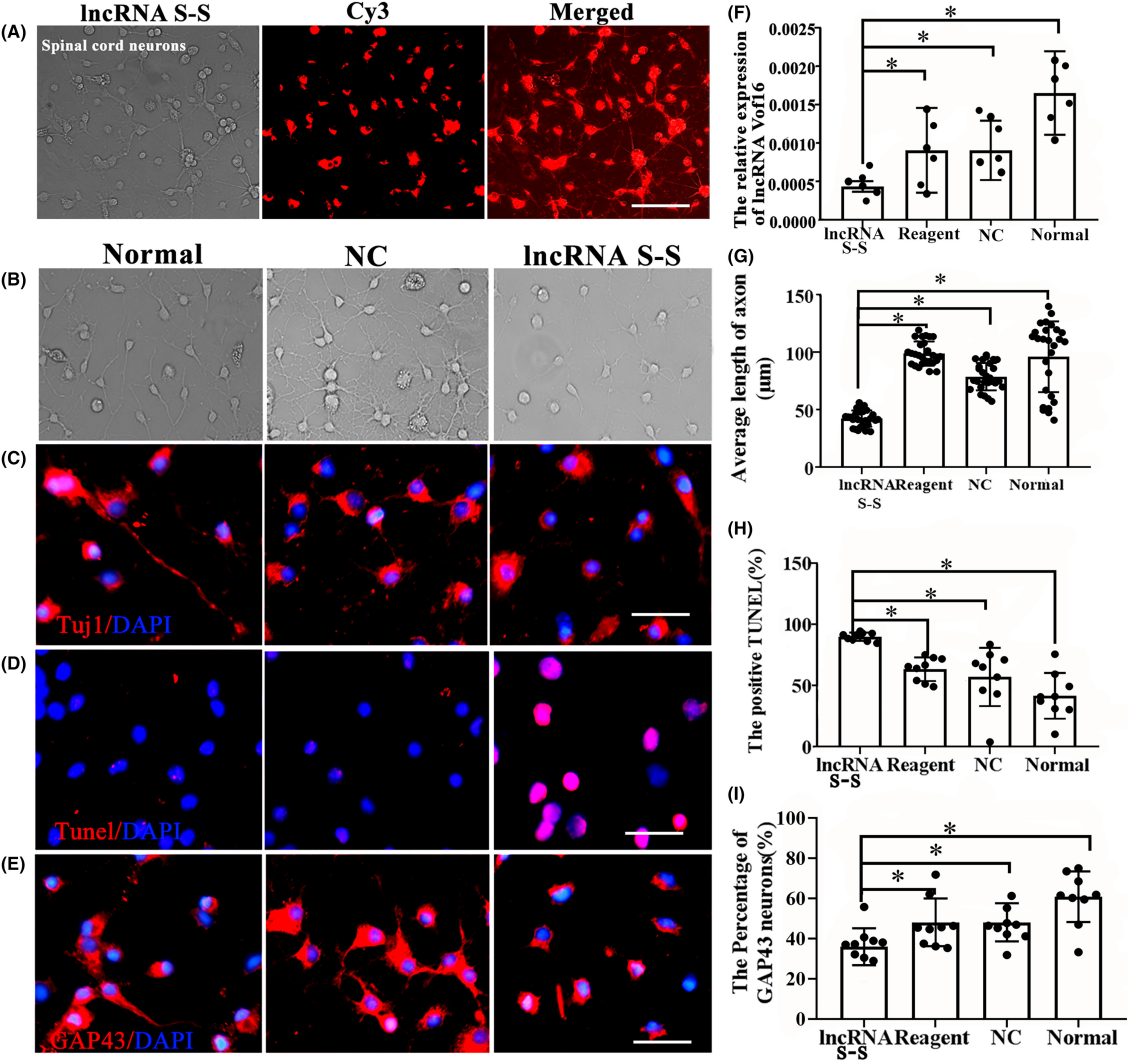
Role of vof16 lncRNA S‐S in the growth of neurite and cell apoptosis in spinal cord neurons. (A) The spinal cord neurons were transfected by CY3 (red)‐labeled lncRNA S‐S and then visualized by fluorescent microscope, *n* = 6/group. Scale bar = 50 μm. (B) The morphology of spinal neurons in the groups of normal, NC, and lncRNA S‐S, respectively, under the light microscope, *n* = 6/group. Scale bar = 50 μm. (C–E) Immunofluorescence staining of Tuj1, TUNEL, and GAP43 in normal, NC as well as lncRNA S‐S groups, respectively, *n* = 6/group. Red fluorescence represents Tuj1, TUNEL, and GAP43 positive cells. Nuclei were stained by DAPI, *n* = 6/group. Scale bar = 50 μm. (F) The relative expression of lncRNA–vof16 among lncRNA S‐S, Reagent, NC, and normal groups, *n* = 6/group. (G–I) Quantitative histograms of neurite length, *n* = 27/group; the percentage of positive TUNEL and GAP43 neurons in lncRNA S‐S compared with Reagent, NC, and normal groups, *n* = 9/group. Data are presented as the means ± SD and analyzed using one‐way ANOVA followed by Student–Newman–Keuls (SNK) post hoc test for multiple comparisons, *p < 0.05. DAPI, 4′,6‐diamidino‐2‐phenylindole; lncRNA S‐S, lncRNA Smart Silencer; NC, negative control; Reagent, transfection reagent; Tuj1, Neuronal Class III β‐Tubulin; TUNEL, Terminal‐deoxynucleotidyl Transferase Mediated Nick End Labeling.

To explore the role of vof16, the primary spinal neurons were transfected with vof16 Ribo™ lncRNA Smart Silencer (lncRNA S‐S) to silence the expression of vof16. Three days after transfection, CY3 (red color) labeled spinal cord neurons confirmed the plasmid of vof16 lncRNA S‐S has been successfully transfected into neurons (Figure 3A). As expected, qRT‐PCR results confirmed that a significant decrease in expression of lncRNA vof16 was observed in lncRNA S‐S group compared with that of non‐targeting NC, Regent, and normal groups (Figure 3F, *p* < 0.05). Additionally, to further evaluate the effect of vof16 lncRNA on cell proliferation and neurite outgrowth, we observed the morphology of neurons after lncRNA vof16 silencing under the light microscope and found obvious axon retraction of spinal neurons compared with normal and NC groups (Figure 3B). Meanwhile, immunofluorescence staining of Tuj1 and TUNEL as well as GAP43 were detected in spinal cord neurons in the groups of normal, NC and lncRNA S‐S. As a result, the neurons with lncRNA S‐S had markedly decreased neurite length, increased number of TUNEL+ cells and reduced expression of GAP43, compared with those of the normal and NC groups (Figure 3C–E). Quantitative analysis revealed that the shorter length of axons, the lower percentage of GAP43‐positive neurons as well as more apoptotic cells apoptosis were observed in vof16 lncRNA S‐S group than NC, normal, and regent groups (Figure 3G–I, *p* < 0.05). These results demonstrated that the downregulation of vof16 could inhibit the growth of spinal cord neurons, indicating vof16 is a beneficial factor for spinal cord neurons.

### 3.3 The expression of GAP43 was significantly reduced after downregulation of lncRNA vof16

**FIGURE 4:**
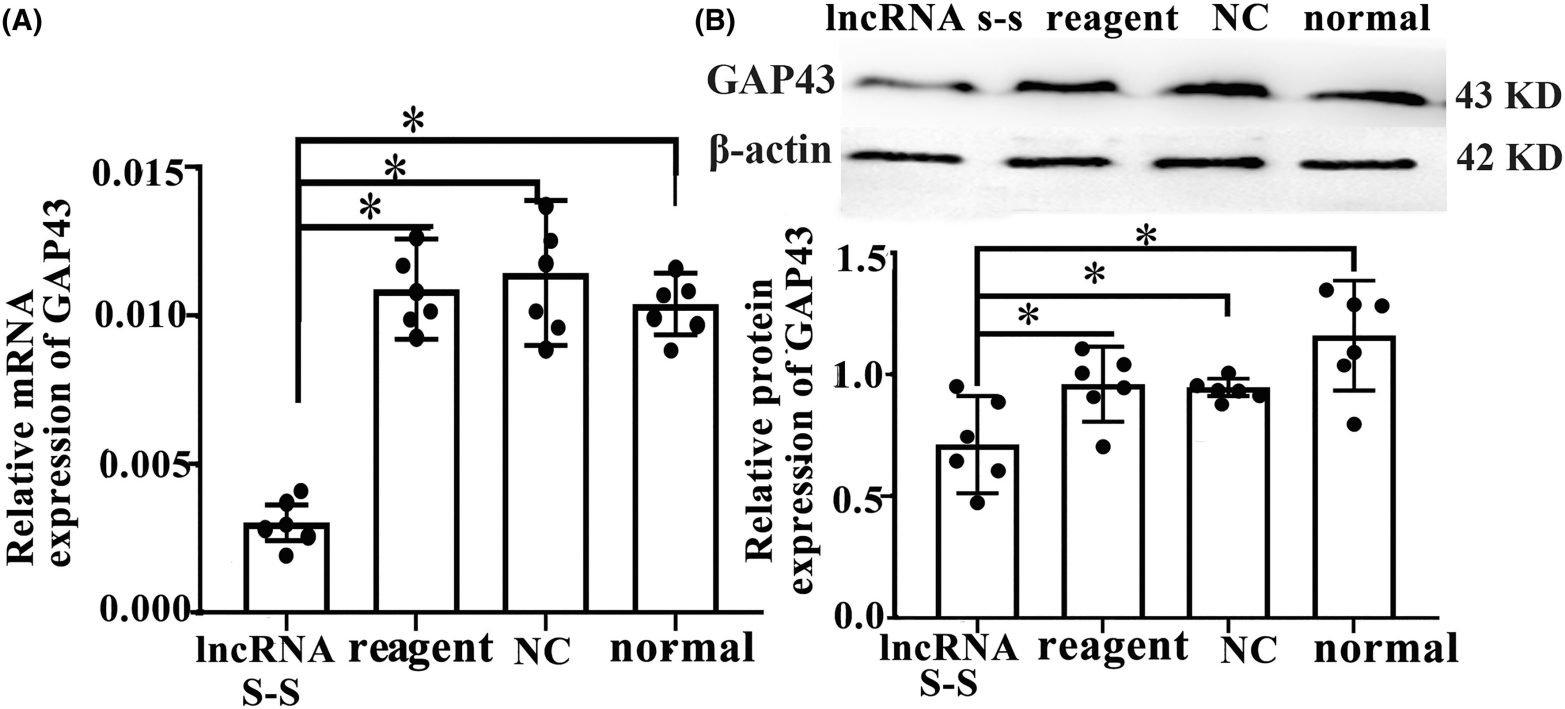
Expression of GAP43 after silencing lncRNA vof16 in spinal cord neurons. (A) The bar chart of relative mRNA expression of GAP43 in the groups of lncRNA S‐S, reagent, NC, and normal, respectively, *n* = 6/group. (B) The western blot stripes and the expression of GAP43 proteins in lncRNA S‐S, reagent, NC, and normal groups, *n* = 6/group. β‐Actin is treated as an internal control. Data are presented as the means ± SD and analyzed by one‐way ANOVA followed by Student–Newman–Keuls (SNK) post hoc test for multiple comparisons, *p < 0.05. lncRNA S‐S, lncRNA Smart Silencer; NC, Negative control; Reagent, Transfection reagent.

In order to further investigate whether the effect of GAP43 on spinal cord neural recovery depends on regulating lncRNA vof16, we evaluated the expression changes of GAP43 after vof16 silenced through Western blot and qRT‐PCR assays. The results showed that the expression level of GAP43 mRNA in the vof16 lncRNA S‐S group was notably lower than the reagent, NC, and normal groups (Figure 4A, *p* < 0.05). Additionally, in agreement with western blot results, a similar decrease in protein expression of GAP43 in the lncRNA S‐S group was revealed compared with that of reagent, NC, and normal groups (Figure 4B, *p* < 0.05). These results ascertain that GAP43 promoting neurological recovery may be associated with lncRNA vof16.

### 3.4 Vof16 knockout decreased axon length and cell migration in spinal cord and cortical neurons

**FIGURE 5:**
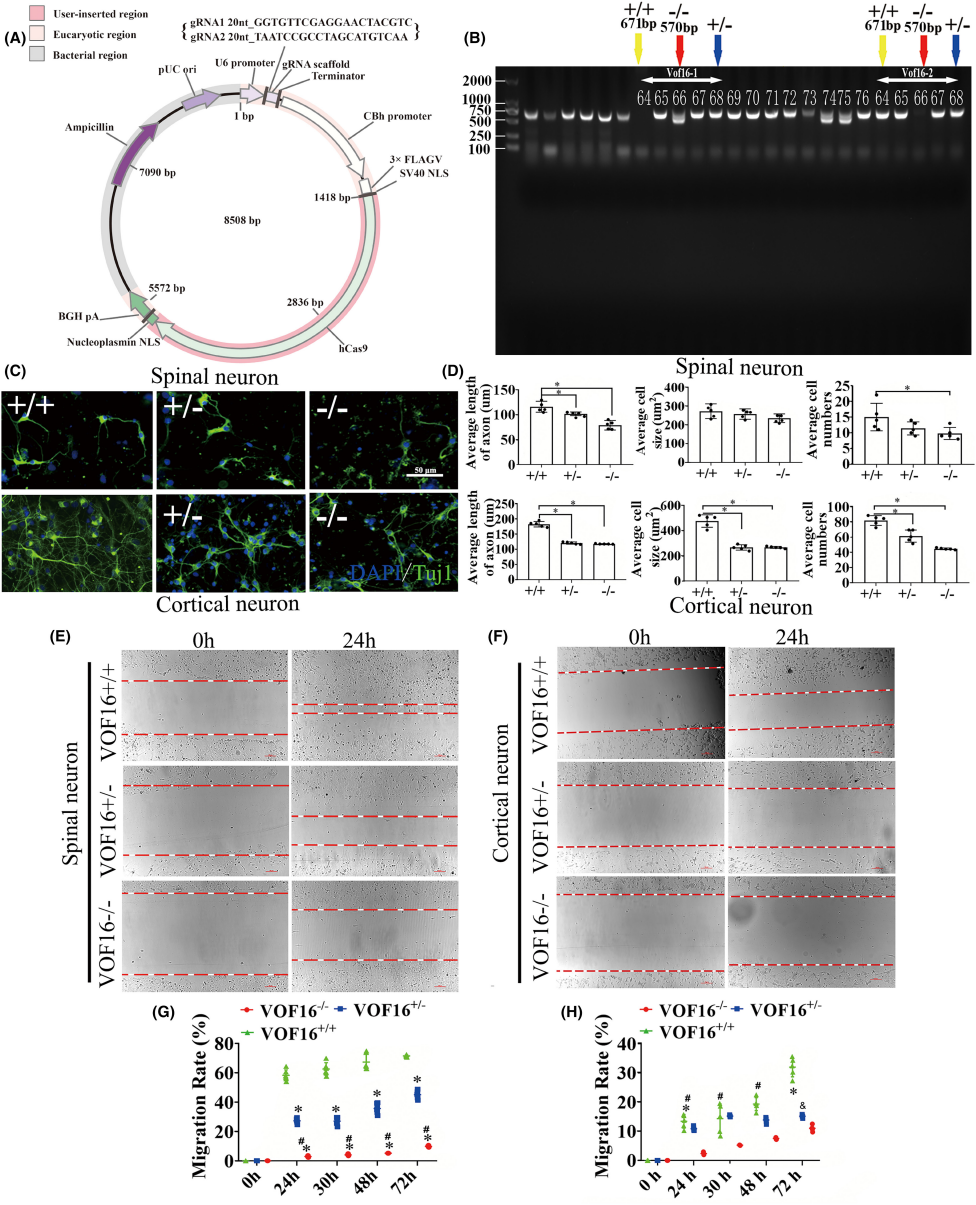
Role of vof16 knockout in the neuron growth and cell migration in spinal cord and cortical neurons. (A) The establishment of vof16 knockout (−/−) recombinant plasmid. (B) Electrophoretic band chart for genotype detection. Yellow arrow represents wild type (+/+) at 671 bp; blue arrow represents heterozygote (+/−); red arrow represents homozygote (−/−) at 570 bp. The markers exhibit 100, 250, 500, 750, 1000, and 2000 bp, respectively. The numbers over each band represent identification numbers of rats in the experiments. (C) Immunofluorescence staining with Tuj1 in spinal and cortical neurons among the groups of vof16^+/+^, vof16^+/−^, and vof16^−/−^, *n* = 3/group. Tuj1 was stained by green fluorescence and the nuclei were stained by DAPI. Scale bar = 50 μm. (D) Quantitative histograms of the neurite length, cell size, and the number of cells in rats with vof16^+/+^, vof16^+/−^, and vof16^−/−^ between spinal cord and cortical neurons, respectively, *n* = 5/group. (E, F) The images of cell migration in these groups at 0 and 24 h in spinal and cortical neurons, *n* = 3/group. Red arrow represents the width of migration. Scale bar = 100 μm. (G) The line chart of migration rate in the groups of vof16^−/−^, vof16^+/−^, and vof16^+/+^ at 0, 24, 30, 48, and 72 h in the spinal cord neurons, *n* = 6/group. *p < 0.05: vof16^−/−^ versus vof16^+/+^; ^#^ *p* < 0.05: vof16^−/−^ versus vof16^+/−^. (H) The rate of migration in cortical neurons at 0, 24, 30, 48, and 72 h among vof16^−/−^, vof16^+/−^ and vof16^+/+^ groups, *n* = 5/group. *p < 0.05: vof16^−/−^ versus vof16^+/+^; ^#^ *p* < 0.05: vof16^−/−^ versus vof16^+/−^; ^&^ *p* < 0.05: vof16^+/−^ versus vof16^+/+^ vof16. Data are presented as the means ± SD and analyzed by one‐way ANOVA followed by Student–Newman–Keuls (SNK) post hoc test for multiple comparisons. DAPI, 4′,6‐diamidino‐2‐phenylindole; Tuj1, Neuronal Class III β‐Tubulin; vof16^−/−^, the rats with vof16 knockout; vof16^+/−^, the rats with vof16 homozygote; vof16^+/+^, the rats with wild type.

To further verify the function of vof16, vof16 knockout (−/−) rats were constructed in Cyagen Biosciences (Cyagen, Guangzhou, China) through CRISPR/CAS9 technology. Briefly, two single gRNA (sgRNA) action targets for vof16 gene were designed. The rat vof16 gene (Gene ID: 259227) is located on rat chromosome 8, and the vof16 (GCTGAAGGAACTTTTTGTC‐GCCTGGCAGATAAAAAAAAA) was chosen as target site. Meanwhile, gRNA and mRNA generated by in vitro transcription were injected into fertilized eggs for production of knockout rats (Figure 5A). Then founders were genotyped by PCR gel electrophoresis: wild type (+/+), homozygote (−/−), and heterozygote (+/−) offspring (Figure 5B). As shown, immunofluorescence staining of Tuj1 was implemented to detect the neuron growth in the condition of vof16^+/+^, vof16^+/−^, and vof16^−/−^, which demonstrated that spinal cord and cortical neurons were obviously retracted after vof16 knockout (Figure 5C). Meanwhile, quantitative analysis of the length of axon in both spinal cord and cortical neurons revealed that they were notably shortened in the vof16^−/−^ and vof16^+/−^ groups compared with that in vof16^+/+^ group (Figure 5D, *p* < 0.05). A similar trend was revealed in cell size and the number of cortical neurons. However, there was no significant difference in the cell size of spinal cord neurons among vof16^+/+^, vof16^+/−^, and vof16^−/−^ groups. Moreover, the number of survived spinal neurons from the vof16^−/−^ rats was much less than that of vof16^+/+^ rats (Figure 5D, *p* < 0.05). In addition, cell migration ability was also measured to assess the role of vof16 in neuroprotective function. The results showed that after the depletion of vof16, the distance of cell migration was much shorter than vof16^+/+^ cells at 24 h in both spinal and cortical neurons (Figure 5E,F). Simultaneously, the migration rate in vof16^−/−^ group was distinctly reduced in spinal neurons when compared with vof16^+/+^ and vof16^+/−^ groups at 12, 30, 48, and 72 h (Figure 5G, *p* < 0.05). In cortical neurons, the rate of migration was significantly decreased in vof16^−/−^ group, as compared to vof16^+/−^ group at 24, 30, and 48 h and to vof16^+/+^ group at 24 and 72 h, respectively (Figure 5H, *p* < 0.05). All of these concluded that vof16 is a beneficial factor for cell proliferation, neurite growth, and cell migration.

### 3.5 Vof16 knockout inhibited spinal nerve growth and motor functional recovery

**FIGURE 6:**
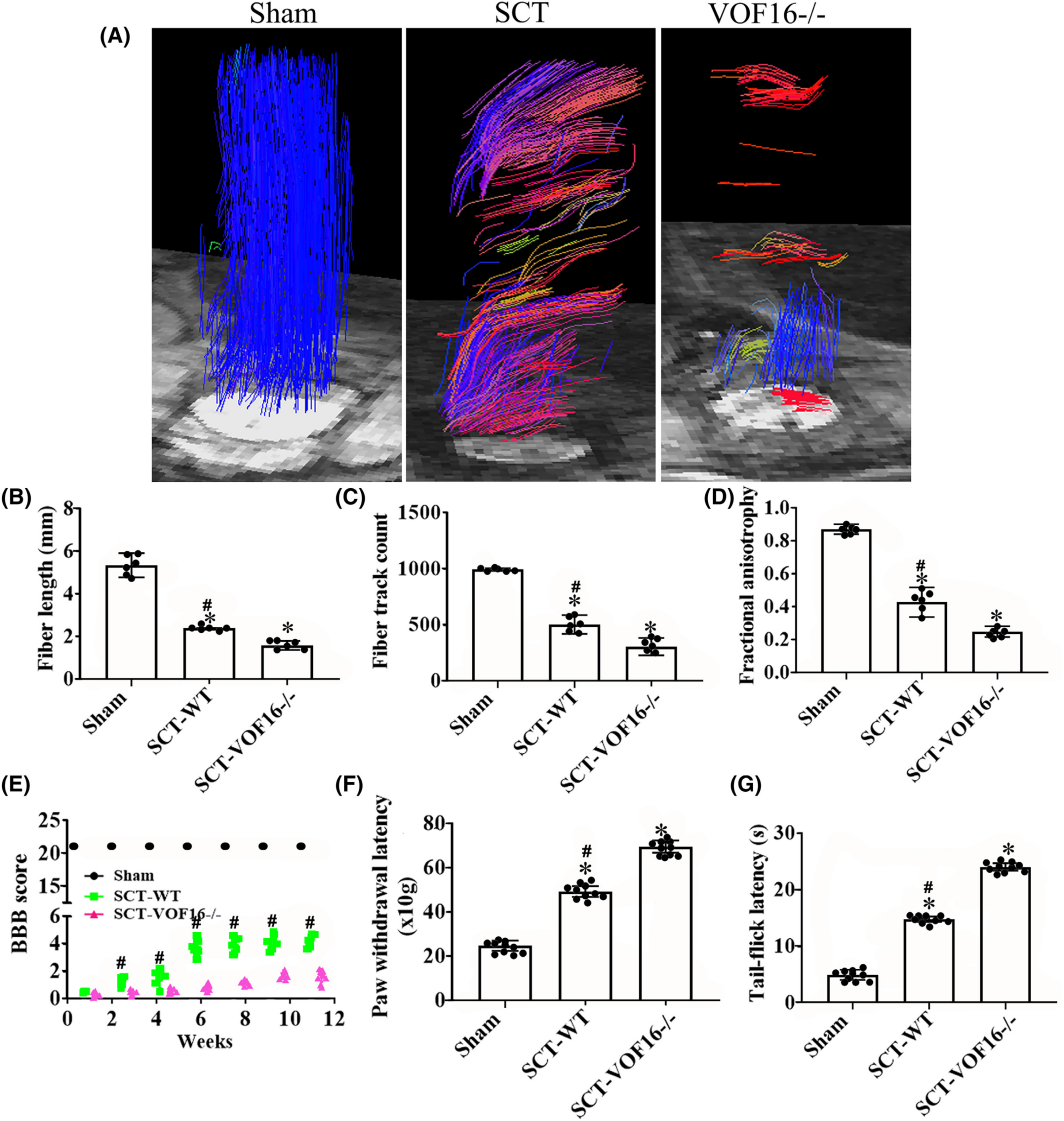
Role of vof16 knockout in the spinal nerve growth after SCT. (A) The DTI scanning images of spinal cord reconstruction in the rats of sham, SCT, and vof16^−/−^ groups, *n* = 6/group. The blue represented the longitudinal fibers, the green and red represented the transverse fibers. (B–D) The bar chart for the quantitative analysis of fiber length, fiber track count, and fractional anisotrophy in the groups of sham, SCT‐WT, and SCT‐vof16^−/−^, *n* = 6/group. (E) BBB score among these three groups at 1, 2, 4, 6, 8, 10, and 12 weeks for motor function, *n* = 10/group. (F, G) Paw withdrawal latency and tail‐flick latency in the sham, SCT‐WT and SCT‐vof16^−/−^ rats, *n* = 10/group. Data are presented as the means ± SD and analyzed by one‐way ANOVA followed by Student–Newman–Keuls (SNK) post hoc test for multiple comparisons. *p < 0.05: SCT‐WT and SCT‐vof16^−/−^ versus sham; ^#^ *p* < 0.05: SCT‐WT versus SCT‐vof16^−/−^. BBB, Basso‐Beattie‐Bresnahan; DTI, Doppler tissue imaging; SCT, spinal cord transection; SCT‐vof16^−/−^, the vof16 knockout rats subjected to spinal cord transection; SCT‐WT, the rats subjected to spinal cord transection.

We used DTI to track spinal cord damage and regeneration of rats in the Sham, SCT and vof16^−/−^ groups at 12 weeks. Different colors were used to track the direction of fiber orientations. The blue represented the longitudinal fibers, the green and red represented the transverse fibers. The DTI images showed that the nerve fibers in the sham group were intact, the blue nerve fibers in SCT‐WT group were interrupted and the many red and green fibers were observed. In addition, the nerve fibers in SCT‐vof16^−/−^ group were poorly reconstructed relative to that in the SCT‐WT group (Figure 6A). The quantitative analysis exhibited that the fiber length and fiber track count of SCT rats were obviously reduced as compared to the sham group, and the decrease in vof16^−/−^ group was significantly lower than that in SCT‐WT group (Figure 6B,C, *p* < 0.05). Fractional anisotropy (FA) values of the spinal segment at the central surgical site demonstrated slight tissue regeneration in SCT‐WT rats, while the SCT‐vof16^−/−^ group showed no signs of regeneration (Figure 6D, *p* < 0.05). These results revealed that vof16 knockout could hamper the axon regeneration in the spinal cord. Furthermore, the motor function of each group was also examined by BBB scores. As a result, the SCT‐vof16^−/−^ rats with has evidently less BBB scores than that of SCT‐WT rats at 1, 2, 4, 6, 8, 10, and 12 weeks, respectively (Figure 6E, *p* < 0.05). Moreover, in order to detect the sensory function, we performed the Paw withdrawal threshold and Tail‐flick latency tests, which revealed that rats suffered SCT significantly spent significantly more time compared with sham‐operated rats (Figure 6F,G, *p* < 0.05), and the SCT‐vof16^−/−^ rats exhibited longer latency as compared to SCT‐WT group (Figure 6F,G, *p* < 0.05). These findings indicate that the deletion of vof16 could weaken spinal nerve growth and hamper motor and sensory functional recovery after SCT.

### 3.6 GAP43 promoted spinal nerve growth after SCT by regulating the vof16‐miRNA‐185‐5p network

**FIGURE 7:**
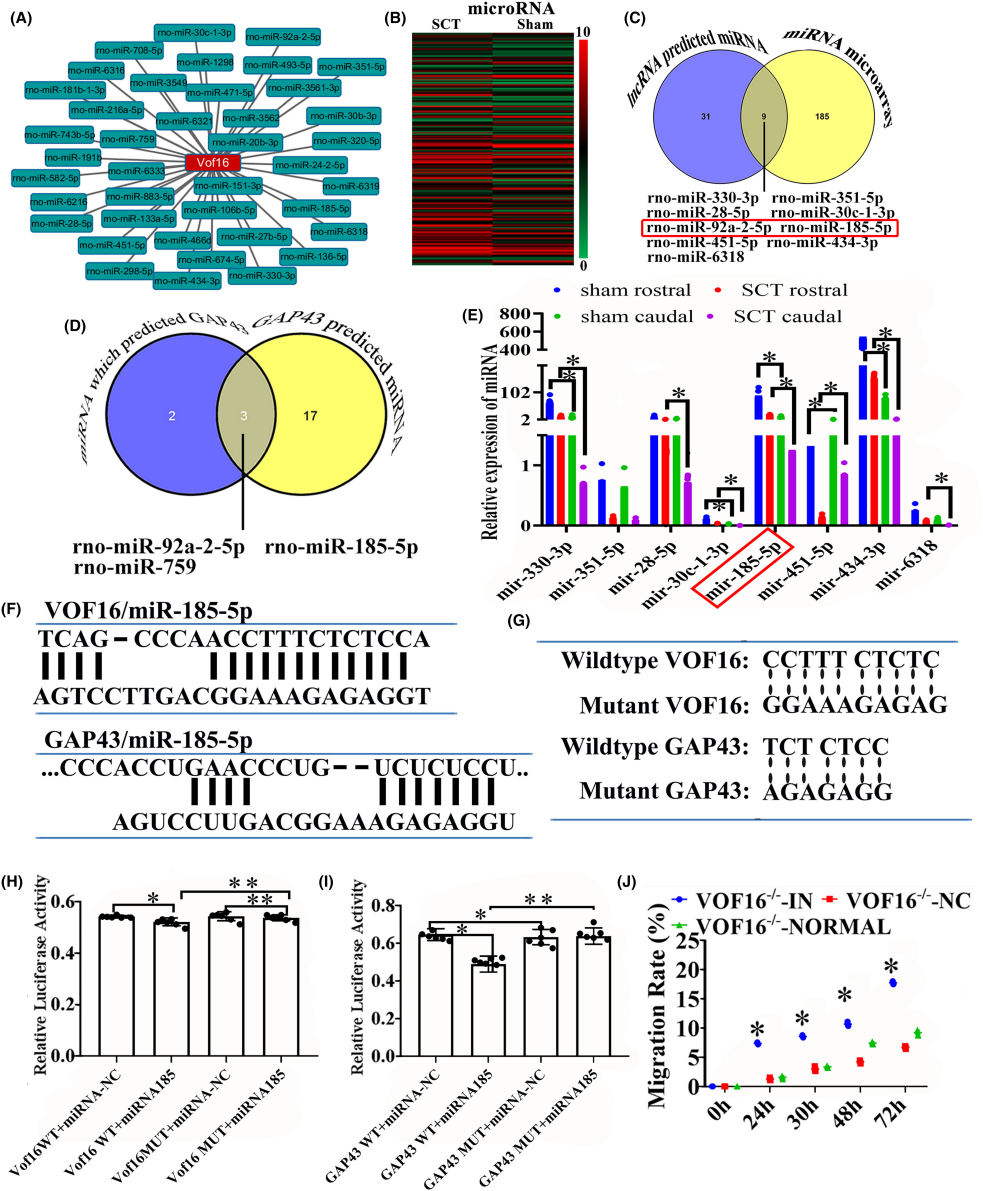
Regulatory relationship among vof16, miR‐185‐5p, and GAP43 in complementary binding nucleotide sequences. (A) The network chart of vof16 with diverse miRNAs. (B) The heat map of miRNA microarray between sham and SCT groups, *n* = 6/group. Red indicates high expression and green for low expression. (C) Intersection of lncRNA‐predicted miRNAs and miRNA microarray to find the miRNAs that are the most relevant to vof16. (D) The cross‐matching chart of miRNA‐predicted GAP43 and GAP43‐predicted miRNAs to screen the miRNA that is closely related to GAP43. (E) The relative repression of miRNA‐330‐3p, miR‐351–5p, miR‐28‐5p, miR‐30c‐1‐3p, miR‐185‐5p, miR‐451–5p, miR‐434‐3p, and miR‐6318 in the groups of sham and SCT at rostral and caudal, *n* = 6/group. (F) The binding sites between vof16, miR‐185‐5p, and GAP43, miR‐185‐5p by RNA22 software and target scan, respectively. (G) The mutant sequences designed for vof16 and GAP43 3′UTR in luciferase reporter assay to effectively abrogate the binding of vof16 and GAP43 to miR‐185‐5p. (H) Relative luciferase activity in vof16‐WT + miRNA‐NC, vof16‐WT + miRNA185, vof16‐MUT + miRNA‐NC, and vof16‐MUT + miRNA185 groups, *n* = 6/group. (I) Relative luciferase activity in the groups of GAP43‐WT + miRNA‐NC, GAP43‐WT + miRNA185, GAP43‐MUT + miRNA‐NC, and GAP43‐MUT + miRNA185 groups, *n* = 6/group. (J) The line chart of migration rate in vof16^−/−^ + IN, vof16^−/−^ + NC and vof16^−/−^ + normal groups at 0, 24, 30, 48, and 72 h in cortical neurons. Data are presented as the means ± SD and analyzed by one‐way ANOVA followed by Student–Newman–Keuls (SNK) post hoc test for multiple comparisons, *p < 0.05; **p < 0.01. IN, miRNA‐185‐5P‐inhibitor; MUT, mutant; NC, negative control; SCT, spinal cord transection; WT, wild type.

The ceRNA analysis was applied for exploring the relationship among lncRNA, miRNA and mRNA and predicting the target candidates of vof16. Thus, Miranda and TargetScan software were carried out and there are 31 potential miRNAs of vof16 stood out (Figure 7A). Additionally, miRNA microarray analysis showed that 185 miRNAs were differentially expressed in rat spinal cord from SCT and sham groups (Figure 7B), and 9 miRNAs out of 185 differentially expressed miRNAs were determined to be regulated by vof16 by the target candidate interaction analysis (Figure 7C). Furthermore, miRNAs which regulated GAP43 were predicted through the TargetScan and miRDB software and Venny website, and the results demonstrated that three miRNAs might regulate the expression of GAP43: miR‐92A‐2‐5P, miR‐185‐5p, and miR‐759 (Figure 7D). To confirm the expression changes in these miRNAs after SCT, qRT‐PCR was employed to quantify their levels between sham and SCT groups in rostral and caudal segments to the transection site. The results showed that SCT significantly decreased the expression of miR‐185‐5p both in rostral and caudal segments compared with the sham group, respectively (Figure 7E, *p* < 0.05). Additionally, to further verify whether vof16 shares regulatory miR‐185‐5p with GAP43, RNA22, and TargetScan were used to predict the binding site between vof16 and miR‐185‐5p, GAP43, and miR‐185‐5p, which was identified in the 3′‐untranslated region (UTR) of vof16 and GAP43 (Figure 7F). Moreover, to ascertain whether this observed effect depends on their regulation of the vof16 and GAP43 3′UTR, we constructed luciferase reporters containing vof16 (vof16‐WT, vof16‐mutant) and GAP43 3′UTR (GAP43‐WT, GAP43‐mutant). Luciferase plasmids (vof16‐WT, vof16‐mutant, GAP43‐WT, GAP43‐mutant) were transfected into the 293T cell clones. As a result, in cells transfected with plasmids containing the vof16‐WT and GAP43‐WT (Figure 7G), the relative luciferase activity was markedly decreased after treatment with miR‐185‐5p, whereas the inhibitory effects of miR‐185‐5p on vof16‐WT and GAP43‐WT were abolished in the plasmid containing the mutants of vof16 as well as GAP43 (Figure 7H,I, *p* < 0.05, *p* < 0.01). Furthermore, we evaluated the rate of cell migration in cortical neurons and found that the lower migration rate in vof16^−/−^ group was obviously rescued after the miR‐185‐5p inhibitor added at 24, 30, 48, and 72 h (Figure 7J, *p* < 0.05). These results implied the important role of vof16 in modulating GAP43 by competitively binding more miR‐185‐5p than GAP43 could bind after SCT.

### 3.7 Inhibition of miR‐185‐5p increased neurite growth regulated by GAP43 in spinal cord neurons

**FIGURE 8:**
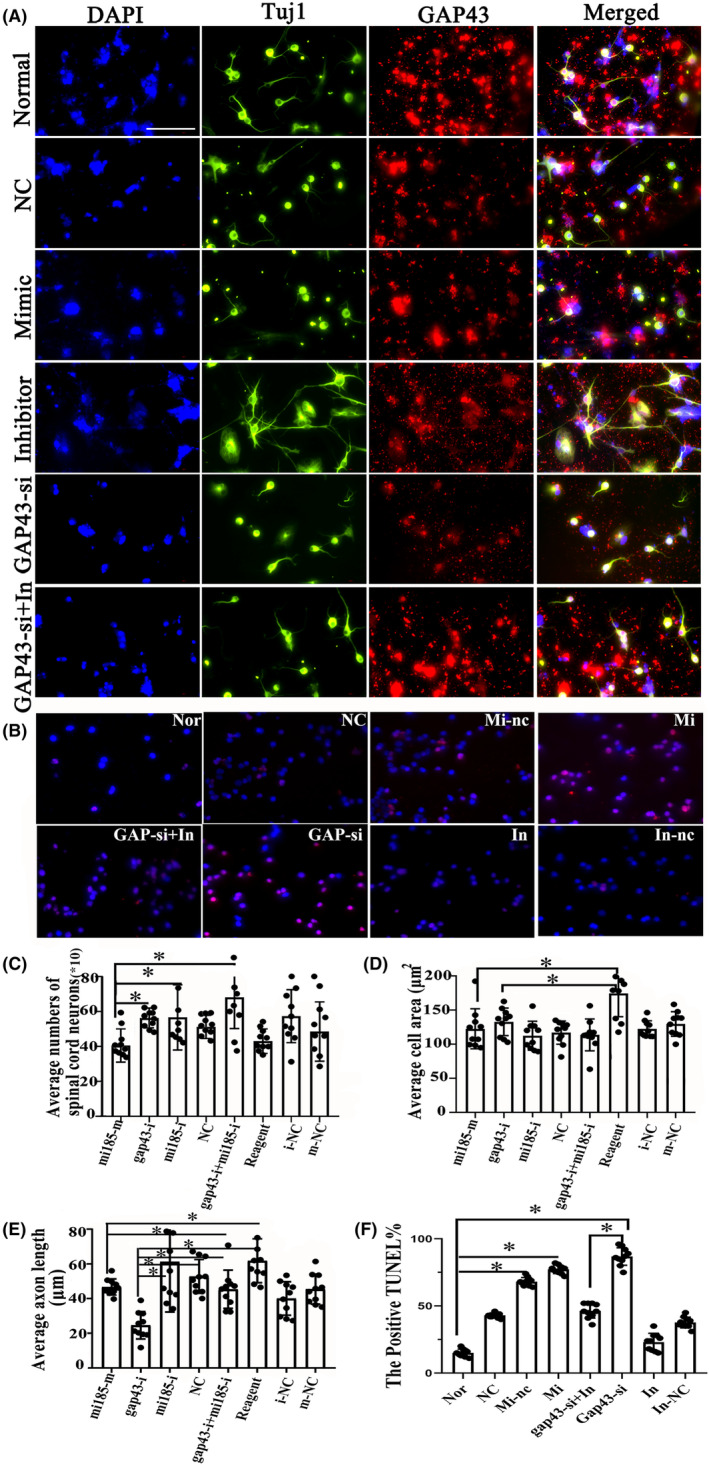
Role of miR‐185‐5p mimics and inhibitor on the neurite outgrowth and cell apoptosis in spinal cord neurons. (A) Immunofluorescence double staining with Tuj1 and GAP43 in the spinal cord neurons in the groups of normal, NC, mimic, inhibitor, GAP43‐si and GAP43‐si + inhibitor, *n* = 10/group. Green fluorescence represents the Tuj1 positive cells, and the nucleus is stained by DAPI. GAP43 was stained by red fluorescence, *n* = 10/group. Scale bar = 50 μm. (B) Immunofluorescence staining with TUNEL in the Normal, NC, Mi‐nc, Mi, GAP43‐si + In, GAP43‐si, In and In‐nc groups in the spinal cord neurons. Apoptotic cells are stained by red color, and the nucleus is stained by DAPI. Scale bar = 50 μm. (C–F) Quantitative histograms of neurons amount, average cell area, axon length as well as the percentage of positive TUNEL among these groups, *n* = 10/group. Data are presented as the means ± SD and analyzed by one‐way ANOVA followed by Student–Newman–Keuls (SNK) post hoc test for multiple comparisons, *p < 0.05. GAP43‐si, GAP43 silencing; In, inhibitor; Mi, miR‐185‐5p‐mimic; NC, negative control; Nor, normal; Tuj1, Neuronal Class III β‐Tubulin; TUNEL, Terminal‐deoxynucleotidyl Transferase Mediated Nick End Labeling.

Immunofluorescence double staining of TUJ1 and GAP43 was performed to determine the spinal cord neurons outgrowth after administration of miR‐185‐5p‐mimic/inhibitor, GAP43‐si (provided by RiboBio, Guangzhou, China) (Figure 8A). In addition, for the rescue experiment, we transfected the miR‐185‐5p‐inhibitor treated cells with GAP43‐si (Figure 8A). As a result, inhibiting miR‐185‐5p contributed to better growth of neurons, accompanied with more expression of GAP43. However, neuronal damage was caused in cells containing miR‐185‐5p‐mimic and GAP43‐si. Interestingly, miR‐185‐5p‐inhibitor could obviously counteract GAP43‐si to induce the decreased number of neurons (Figure 8A). Meanwhile, quantification histograms displayed that the number of spinal cord neurons in miR‐185‐5P‐mimic was notably reduced compared with miR‐185‐5p‐inhibitor (Figure 8C, *p* < 0.05). The cell area and axon length in the groups of miR‐185‐5p‐mimic and GAP43‐si were significantly decreased, as compared to the reagent group (Figure 8D,E, *p* < 0.05), whereas, the shortened neurites after GAP43 silencing were abolished by miR‐158‐5p‐inhibitor (Figure 8E, *p* < 0.05). These revealed a neutralizing relationship between GAP43 and miR‐185‐5p in axon growth. Furthermore, the cell apoptosis was also evaluated by immunofluorescent staining of Tunel in these groups, which exhibited that more cell apoptosis was observed in miRNA‐185‐5p‐mimic and GAP43‐si groups when compared to NC group, while miR‐185‐5p‐inhibitor did the opposite (Figure 8B). These findings revealed that downregulating miR‐185‐5p could promote axon growth via modulating GAP43.

### 3.8 MiR‐185‐5p knockout enhanced cell growth and migration and improved motor and sensory functions after silencing GAP43

**FIGURE 9:**
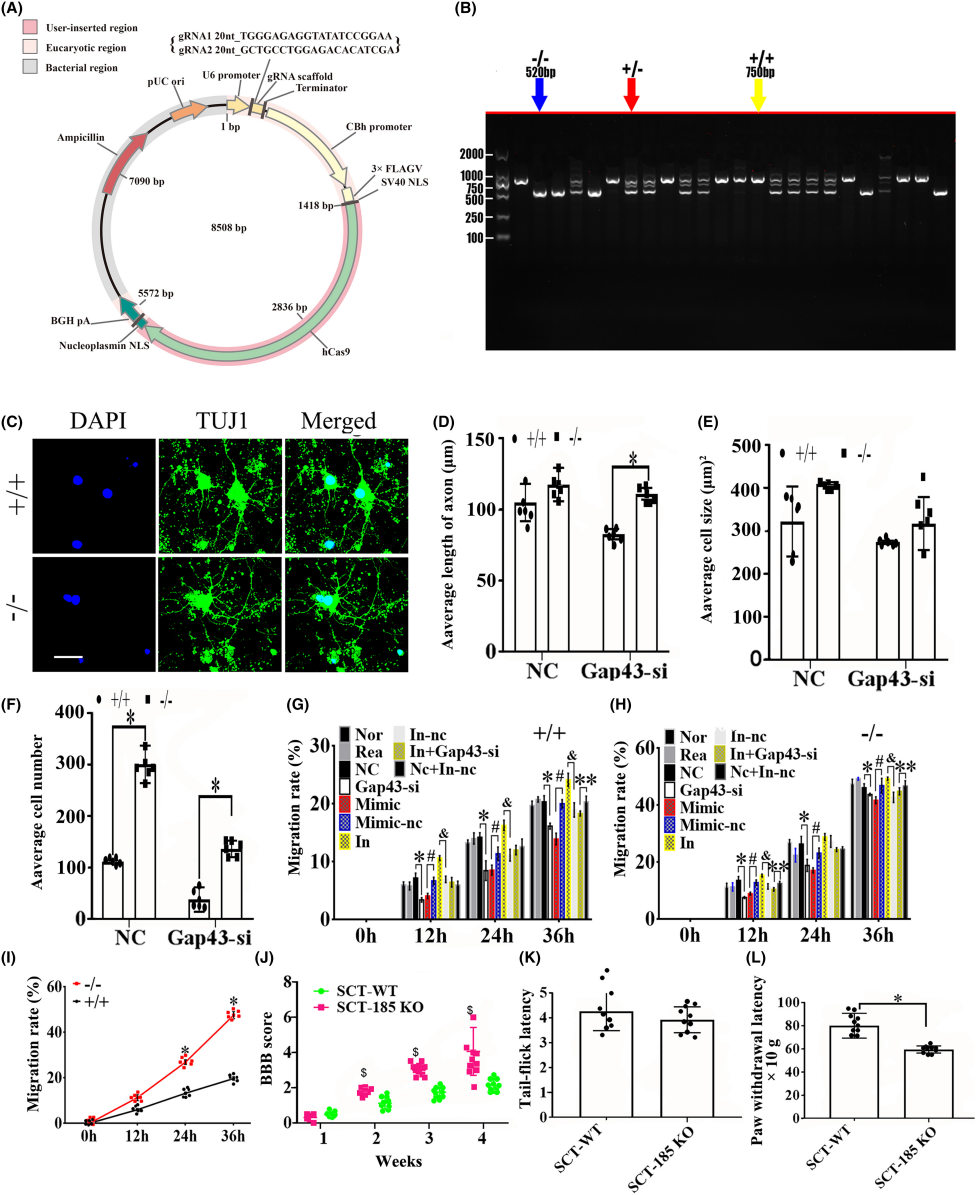
Effect of miR‐185‐5p knockout in spinal cord neurons and recovery after injury. (A) The establishment of miR‐185‐5p knockout (−/−) recombinant plasmid. (B) The electrophoretic band chart for genotype detection. Blue arrow represents knockout (−/−) rats at 520 bp; red arrow represents heterozygote rats; yellow arrow represents wild‐type (+/+) rats at 750 bp. The markers exhibit 100 bp, 250 bp, 500 bp, 750 bp, 1000 bp, and 2000 bp, respectively. (C) Immunofluorescence staining of Tuj1 between miR‐185‐5p^+/+^ and miR‐185‐5p^−/−^ in spinal cord neurons, *n* = 6/group. The Tuj1‐positive neurons are stained by green fluorescence. Blue represents the nucleus. Scale bar = 50 μm. (D–F) The quantitative analysis of axon length, cell size and cell numbers in NC and GAP43‐si neurons with miR‐185‐5p^+/+^ or miR‐185‐5p^−/−^ *n* = 6/group. (G) The migration rate among Normal, Reagent, GAP43‐si, Mimic, Mimic‐nc, In, In‐nc, In+GAP43‐si and Nc + In‐nc groups in miR‐185‐5p^+/+^ neurons respectively at 0,12, 24, and 36 h, *n* = 6/group. (H) The migration rate in Normal, Reagent, NC and GAP43‐si in miR‐185‐5p^−/−^ neurons at the same time, *n* = 6/group. (I) The migration rate between miR‐185‐5p^+/+^ and miR‐185‐5p^−/−^ groups at 0,12, 24, and 36 h, *n* = 6/group. (J) BBB score of the rats in miR‐185‐5p^+/+^ and miR‐185‐5p^−/−^groups at 1, 2, 3, and 4 weeks after SCT, *n* = 10/group. (K, L) The time of paw withdrawal threshold and tail‐flick latency in the miR‐185‐5p^+/+^ and miR‐185‐5p^−/−^ rats after SCT, *n* = 10/group. Data are presented as the means ± SD and analyzed by one‐way ANOVA followed by Student–Newman–Keuls (SNK) post hoc test for multiple comparisons, *p < 0.05; ^#^ *p* < 0.05; ^&^ *p* < 0.05; **p < 0.01. BBB, Basso‐Beattie‐Bresnahan; DAPI, 2‐(4‐Amidinophenyl)‐6‐indolecarbamidine dihydrochloride; GAP43‐si, silencing GAP43; In, miR‐185‐5p‐inhibitor; Mimic, miR‐185‐5p‐mimic; NC, negative control; Nor, normal; Rea, reagent; SCT, Spinal cord transection; Tuj1, Neuronal Class III β‐Tubulin.

We established the miR‐185‐5p knockout (−/−) recombinant plasmid (Figure 9A). To further investigate whether miR‐185‐5p affects cell growth through functioning together with GAP43, we established the miR‐185‐5p knockout rats through CRISPR/CAS9 technology. The genotype was verified via PCR gel electrophoresis including wild type (+/+), homozygote (−/−), and heterozygote (+/−) off springs (Figure 9B). The immunofluorescence staining of Tuj1 showed the better spinal cord neurons outgrowth in the miR‐185‐5p^−/−^ group than miR‐185‐5p^+/+^ group (Figure 9C). In the spinal cord neurons treated with GAP43‐si, the axon length and the number of cells in miR‐185‐5p^−/−^ group were notably increased, as compared with miR‐185‐5p^+/+^ group (Figure 9D, *p* < 0.05). Moreover, compared with miR‐185‐5p^+/+^ group, the miR‐185‐5p^−/−^ group had a higher cell number (Figure 9F, *p* < 0.05), whereas, the cell size between the two groups has no obvious significance (Figure 9E, *p* > 0.05). The cell migration test revealed that after silencing GAP43, the rate of migration was significantly reduced in miR‐185‐5p^+/+^ neurons compared with NC group at 12, 24, and 36 h (Figure 9G, *p* < 0.05), which corresponded with that in miR‐185‐5p^−/−^ neurons (Figure 9H, *p* < 0.05). Furthermore, compared with mimic‐nc group, the miR‐185‐5p‐mimic group exhibited the lower migration rate. On the contrary, inhibiting miR‐185‐5p could markedly increase the migration ability in WT neurons at 12, 24, and 36 h (Figure 9G, *p* < 0.05). What's more, the rate of migration in miR‐185‐5p‐inhibitor+GAP43‐si group was evidently reduced compared with NC+ inhibitor‐nc group in WT neurons at 36 h (Figure 9G, *p* < 0.05). We also found that miR‐185‐5p^−/−^ could obviously increase the migration rate, as compared with WT groups at 24 and 36 h (Figure 9I, *p* < 0.05). Moreover, the motor and sensory function was also determined by BBB score, Tail‐flick latency, and Paw withdrawal latency in vivo. As a result, the rats with miR‐185‐5p^−/−^ had a higher BBB score than the WT rats at 2, 3, and 4 weeks after SCT (Figure 9J, *p* < 0.05), and the time of paw withdrawal latency was notably reduced in miR‐185‐5p^−/−^ rats, as compared to the rats with WT after SCT (Figure 9L, *p* < 0.05). However, there was no significant difference between WT and miR‐185‐5p^−/−^ groups in tail‐flick latency (Figure 9K, *p* > 0.05). All these findings above suggested that knockout of miR‐185‐5p can protect spinal cord neurons and ameliorate the motor and sensory dysfunction induced by SCT via regulating GAP43.

## 4 DISCUSSION

Previous researches have shown that miRNAs and lncRNAs play important roles in regulatory mechanisms for the treatment of cancers and other diseases. Here, we found that vof16‐miR185‐5p‐GAP43 network is closely related to SCT self‐repair, and simultaneously enhances motor and sensory function after SCT. Knocking out vof16 or GAP43 could inhibit the self‐repair of spinal cord and neurite growth, while miR‐185‐5p knock‐out promoted the axonal growth after SCT. Furthermore, miR‐185‐5p can competitively bind the same regulatory region of vof16 and GAP43. Our study provides a novel regulatory network that functions via a miRNA competitive mechanism mediated by vof16 in SCT self‐repair and axonal growth. It may underlie the feasible foundation for the treatment of SCT in future clinical trials.

The rats subjected to SCT showed deficient motor function with poor recovery in hind limbs in the early phase, whereas the recovery was increased over time, especially at 12 weeks after the SCT, which was accompanied by the increased expression of GAP43. Accumulating evidence has indicated that primitive vertebrates like certain fishes, cyclostomes, amphibians, and mammals such as rats can repair their damaged spinal cords and recover from some function loss after SCI.^40^, ^41^, ^42^, ^43^, ^44^, ^45^ The strategies for functional recovery after SCI with self‐repair capabilities are diverse and mostly based on plasticity within the lower spinal cord segments and axon regeneration.^46^ Meanwhile, neurons transiently upregulate regeneration‐associated genes^47^ and severed axons which are near the lesion may grow sprouts. Spared axons that grow collateral sprouts may contribute to the occasionally observed natural recovery of function.^10^, ^48^ GAP43 is a protein that is studied extensively and has been described for its abundance in axonal growth cones.^49^, ^50^, ^51^ It is used widely as a marker of axonal sprouting.^52^, ^53^ A previous research found that in GAP‐43 overexpressed mice, motor neurons underwent increased axonal sprouting after lesion.^54^ In our study, we observed that the expression of GAP43 was significantly elevated in spinal cord at 12 weeks after SCT when an obvious locomotor function recovery was also observed. Therefore, our results indicated that a higher level of GAP43 is associated with self‐repair and functional recovery after SCT.

Presently, studies have shown that lncRNAs are involved in nervous system development at many levels including neuronal differentiation and functional retention, neuronal cell apoptosis, and brain development.^55^ The expression of a series of lncRNA is altered in SCT rats model, and lncSCIR1 knockdown promotes astrocyte proliferation and migration in vitro.^3^ In addition, it has been proved that inhibition of lncRNA IGF2AS has profound effects on inducing neuronal growth and protecting local‐anesthetic‐induced neurotoxicity in dorsal root ganglion neurons.^56^ By RNA sequencing and GO analysis on the spinal cord of SCT, vof16 was found as one of the most relevant lncRNAs for its differential high expression.^57^ Vof16 was abundant in the hippocampus, the tenia tecta, the piriform cortex, and the area around the aorta.^58^ The expression of vof16 was substantially up‐regulated in the cortex and hippocampus of rats with HI injury,^13^ and its high level has been reported to correlate with neuronal damage and impairment of memory and learning ability.^59^ In our study, silencing (or knockout) lncRNA vof16 inhibited neurite growth and increased cell apoptosis in both spinal cord and cortical neurons in vitro. Meanwhile, vof16 knock out rats showed reduced nerve reconstruction and attenuated motor and sensory functions after SCT in vivo. The present study has revealed a novel function of vof16 in regulating neuronal growth and locomotor functional recovery in the rat model of SCT. Therefore, a series of lncRNAs play important regulatory roles in the pathology of SCI.

Additionally, through PCR verification, Western blot as well as immunofluorescence staining with GAP43, we observed that downregulating vof16 was notable to decrease the mRNA and protein level of GAP43 and inhibited the axon regrowth. Our data in this present study has demonstrated that GAP43 is associated with self‐repair after SCT. Therefore, to explore the relative regulatory mechanism between GAP43 and lncRNA‐vof16 during the process of repairing, we performed double prediction via RNA22 and TargetScan, and combined with luciferase reporter assays. Our results indicated that miR‐185‐5p was closely correlated with vof16 and GAP43 in both regulatory sites and function. In order to further verify the prediction, miR‐185‐5p‐mimic/inhibitor and silencing GAP43 were applied to examine their regulatory relationship in spinal cord neurons, which was also observed in miR‐185‐5p KO rats in vitro and in vivo. We have found that inhibiting miR‐185‐5p could obviously counteract GAP43‐si‐induced decrease of neurite growth and increase in cell migration. In addition, miR‐185‐5p KO rats performed better in motor and sensory functions after SCT. Recently, miR‐185 has been reported as a tumor suppressor^60^ and to be down‐regulated in gastric cancer, breast cancer, glioma, clear renal cell carcinoma, and hepatocellular carcinoma.^61^, ^62^, ^63^, ^64^, ^65^ Besides, a previous research further found that the miR‐185‐5p is associated with the lncRNA FOXD2‐AS1 to contribute to colorectal cancer proliferation.^66^ However, its function in the nervous system remains unknown. Regarding the specific mechanism underlying the function of vof16, we further employed bioinformatics to predict entangled factors with vof16 and applied luciferase assay to determine the regulatory relationship among vof16, miR‐185‐5p, and GAP43, which demonstrated that vof16 and GAP43 are both the targets of miR‐185‐5p. MiR‐185‐5p could bind with either vof16 or GAP43. The elevation of GAP43 expression might be attributed to the phenomenon that a lot of miR‐185‐5p is bound and degraded by vof16, thus miR‐185‐5p's binding with GAP43 decreased. Our data indicated that miR‐185‐5p could be a detrimental factor in SCT, and vof16 may function as a ceRNA by competitively binding miR‐185‐5p and modulate GAP43 in the process of self‐recovery after SCT.

## 5 CONCLUSIONS

Our research has revealed that vof16 and miR‐185‐5p are important regulators of GAP43, and vof16 could promote the effects of GAP43 on axonal regrowth after SCT but miR‐185‐5p negatively counteracts vof16 and GAP43. Our study also confirmed the vital role of the vof16‐miR185‐5p‐GAP43 network in regulating self‐repair of SCT injury by promoting the GAP43‐dependent neurite growth. The findings have important implications in developing strategies to improve function after SCI and provide a potential therapeutic target for clinical trials in the future. Certainly, limitations exist in this study since only one sex of the rats (female) was selected in our experiment design, thus the potential sex differences failed to be presented.

## FUNDING INFORMATION

This study was supported by the grant of National Natural Science Foundation Regional Fund of China (No. 82060243), Guizhou Provincial Higher Education Science and Technological Innovation Team (grant number: [2023]072), Guizhou Province Distinguished Young Scientific and Technological Talent Program (grant number: YQK[2023]040) and Collaborative Innovative Center of Chinese Ministry of Education (2020‐39).

## CONFLICT OF INTEREST STATEMENT

The authors declare no conflicts of interest.

## Data Availability

All data are available from the corresponding author on a reasonable request.
